# A microsurgical robot research platform for robot-assisted microsurgery research and training

**DOI:** 10.1007/s11548-019-02074-1

**Published:** 2019-10-11

**Authors:** Dandan Zhang, Junhong Chen, Wei Li, Daniel Bautista Salinas, Guang-Zhong Yang

**Affiliations:** The Hamlyn Centre for Robotic Surgery, Imperial College London, London, SW7 2AZ UK

**Keywords:** Microsurgical robot, Robot operating system (ROS), Master–slave control

## Abstract

**Purpose:**

Ocular surgery, ear, nose and throat surgery and neurosurgery are typical types of microsurgery. A versatile training platform can assist microsurgical skills development and accelerate the uptake of robot-assisted microsurgery (RAMS). However, the currently available platforms are mainly designed for macro-scale minimally invasive surgery. There is a need to develop a dedicated microsurgical robot research platform for both research and clinical training.

**Methods:**

A microsurgical robot research platform (MRRP) is introduced in this paper. The hardware system includes a slave robot with bimanual manipulators, two master controllers and a vision system. It is flexible to support multiple microsurgical tools. The software architecture is developed based on the robot operating system, which is extensible at high-level control. The selection of master–slave mapping strategy was explored, while comparisons were made between different interfaces.

**Results:**

Experimental verification was conducted based on two microsurgical tasks for training evaluation, i.e. trajectory following and targeting. User study results indicated that the proposed hybrid interface is more effective than the traditional approach in terms of frequency of clutching, task completion time and ease of control.

**Conclusion:**

Results indicated that the MRRP can be utilized for microsurgical skills training, since motion kinematic data and vision data can provide objective means of verification and scoring. The proposed system can further be used for verifying high-level control algorithms and task automation for RAMS research.

## Introduction

Microsurgery encompasses ophthalmic, ENT, brain surgeries and other surgical tasks involving microscale precisions. Microsurgical procedures require operating field magnification through a microscope to allow for delicate tissue manipulation for targets such as small blood vessels, nerves and other small, delicate structures. The main challenge for microsurgery is being able to perform tasks beyond the level of human perception and dexterity [1]. The poor sensory feedback to human operators brings challenges to microscale manoeuvres in a confined environment [2]. Other difficulties include sophisticated teleoperation mapping [3], the small field of view provided by the microscope [4], occlusions, instrument collision and a lack of tactile and haptic feedback.

To address the challenges mentioned above, the last decade has seen many emerging technologies for improved robot-assisted microsurgery. Despite the clinical success of systems such as the da Vinci system (Intuitive Surgical, Inc., USA) [5], the current platforms are not catered for complex microsurgical tasks. They also lack specific microsurgical tools. The JPL RAMS Workstation has six serial revolute joints [6]. However, it could not hold and operate a microsurgical needle, since it can only be used as a supportive platform for microsurgery. The intra-ocular dexterity robot (IODR), a hybrid two-arm Stewart platform, is equipped with a 2 degrees-of-freedom (DoF) microsurgical tool for ocular surgery [7]. Other emerging microsurgical platforms for ocular surgery include the multi-arm stabilizing micromanipulator [8] and the Japanese ocular robot of Ueta [9]. As for ENT surgery, the RobOtol [10] and the system from Bern University [11] have shown promising results.

In practice, a versatile research platform can assist the training of microsurgical skills and accelerate the development of RAMS. For example, further research can be explored in the area of semi-autonomous teleoperation, including supervisory control, shared control and other co-robotic methods. The availability of open-source research platforms such as the Raven II robot [12] and the da Vinci Research Kit (dVRK) [13] set good examples for developing common research platforms for MIS. However, the precision of these two robots is at mm level, which is not suitable for RAMS.

In recent years, the Robot Operating System (ROS) has become widely adopted among both the research and industrial communities. It provides many shared libraries and utility tools, avoiding low-level repetition and bringing more opportunities for collaboration. Moreover, it enables inter-process communication across hardware platforms [14]. Therefore, it is worthwhile to develop a ROS-based microsurgical robotic platform for research or training.

Since master–slave control has been implemented in most of the current surgical robotic platforms for RAMS, the control interface is essential for safe and effective teleoperation [15]. Most of the slave robots are normally designed based on specific requirement of surgery, while a master robot design would need careful consideration of ergonomics. Therefore, most of the master and slave robots are heterogeneous. The joint-to-joint mapping is natural and intuitive; however, it is not applicable to heterogeneous master–slave robot. To this end, an intuitive master–slave control interface should be explored to ensure the teleoperation efficiency.

In this paper, a microsurgical robot research platform (MRRP), with sub-micron-scale positioning accuracy, is developed for RAMS. It can also be utilized for microsurgical skills training. Experiments are conducted to verify the usability of the platform and assess the effectiveness of the proposed hybrid interface for microsurgical robot control.

The details of the proposed framework are described as follows: Firstly, the system overview of the ROS-based MRRP is introduced in “System overview” section. A master–slave control hybrid interface is presented and analysed in “A novel hybrid interface” section. Finally, discussions are presented in “Discussion” section, while conclusions are drawn in “Conclusions and future works” section.

## System overview

In this section, we presented an overview of the MRRP by detailing the construction of the reconfigurable hardware system and the ROS-compatible software architecture.

### Hardware system

#### The slave robot

**Fig. 1:**
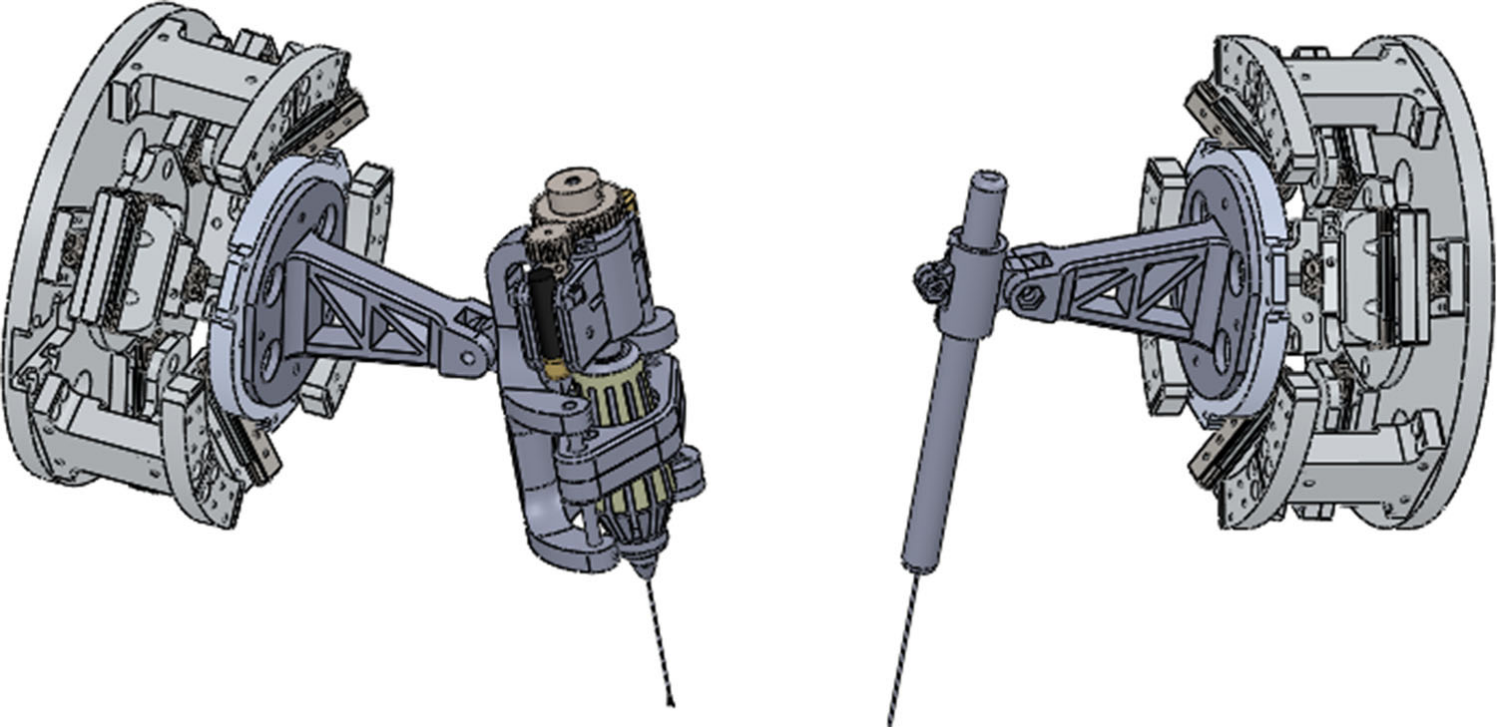
The CAD model of the slave robot for MRRP. A micro-25ga Tano Polisher is mounted on the right manipulator, while a 25ga Maculorhexis Forceps is mounted on the left manipulator

The CAD model of the slave robot for MRRP is shown in Fig. 1. The slave robot consists of two manipulators, which can be used for bimanual microsurgical operations. Different microsurgical tools can be mounted on the stage of the manipulators for different applications. For example, microforceps can be utilized for membrane lifting, while micropolishers can be used for neuroepithelial membrane polishing. Other microsurgical tools include microneedles, microdropper and microblunt tips.

**Fig. 2:**
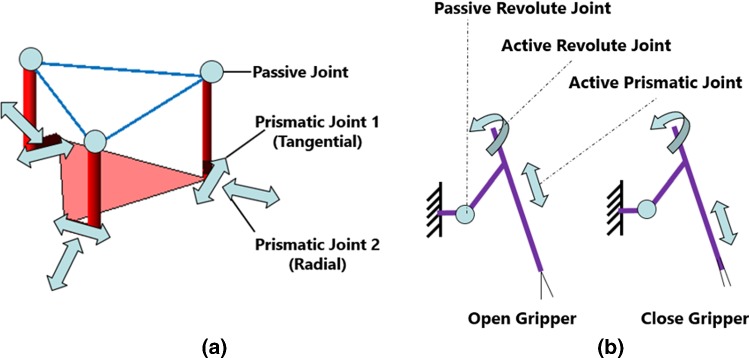
Kinematic structure of the slave robot of MRRP and the sketch for the motorized microsurgical tool. **a** The kinematic structure of the slave robot; **b** the sketch and working mechanism illustration for the motorized microsurgical tool

The manipulators of the slave robot are developed based on two 6-DoF positioning stages SmarPod (SmarAct, Germany), which can be regarded as two manipulators. The hexapod-like SmarPod incorporates parallel kinematics, which has a higher control accuracy and rigidity than the common used platforms using serial kinematics. It is compact and lightweight. A schematic illustration of the kinematic structure for SmarPod is shown in Fig. 2a. The robot consists of three separate serial chains, each of which has an identical kinematic configuration. Each serial chain has two prismatic joints that are formed by a tangential positioner and a radial positioner, respectively. The positioners are based on linear crossed-roller slides, which can ensure high positioning accuracy and resolution. The passive joint is constructed by a passive guideway and a passive ball joint. All actuators are stick-slip piezoinertia drives, with sub-micrometre resolution. By moving all the positioners, 6-DoF can be achieved for each manipulator. The smallest increment of the position is 1 nm, while the smallest increment of orientation is $1~{\upmu }$ rad. The range of the motion in the *X*, *Y* and *Z* axes is 20 mm, 20 mm and 10 mm, respectively. The rotational range is $21^\circ$, $24^\circ$ and $38^\circ$, respectively.

#### The microsurgical tools

The microsurgical platform can be reconfigured. The microsurgical tool modules are replaceable. For example, adding or removing surgical tools or even splitting the system into multiple independent setups can be achieved based on different microsurgical training targets or research requirements.

The sketch of the motorized microforceps is illustrated in Fig. 2b. The gripping motions are realized by an active prismatic joint. The roll motions of the microforceps are provided by another motor, which can be regarded as an active revolute joint. A passive revolute joint is utilized to adjust the relative angle between the central line of the microsurgical tool and that of the slave robot.

**Fig. 3:**
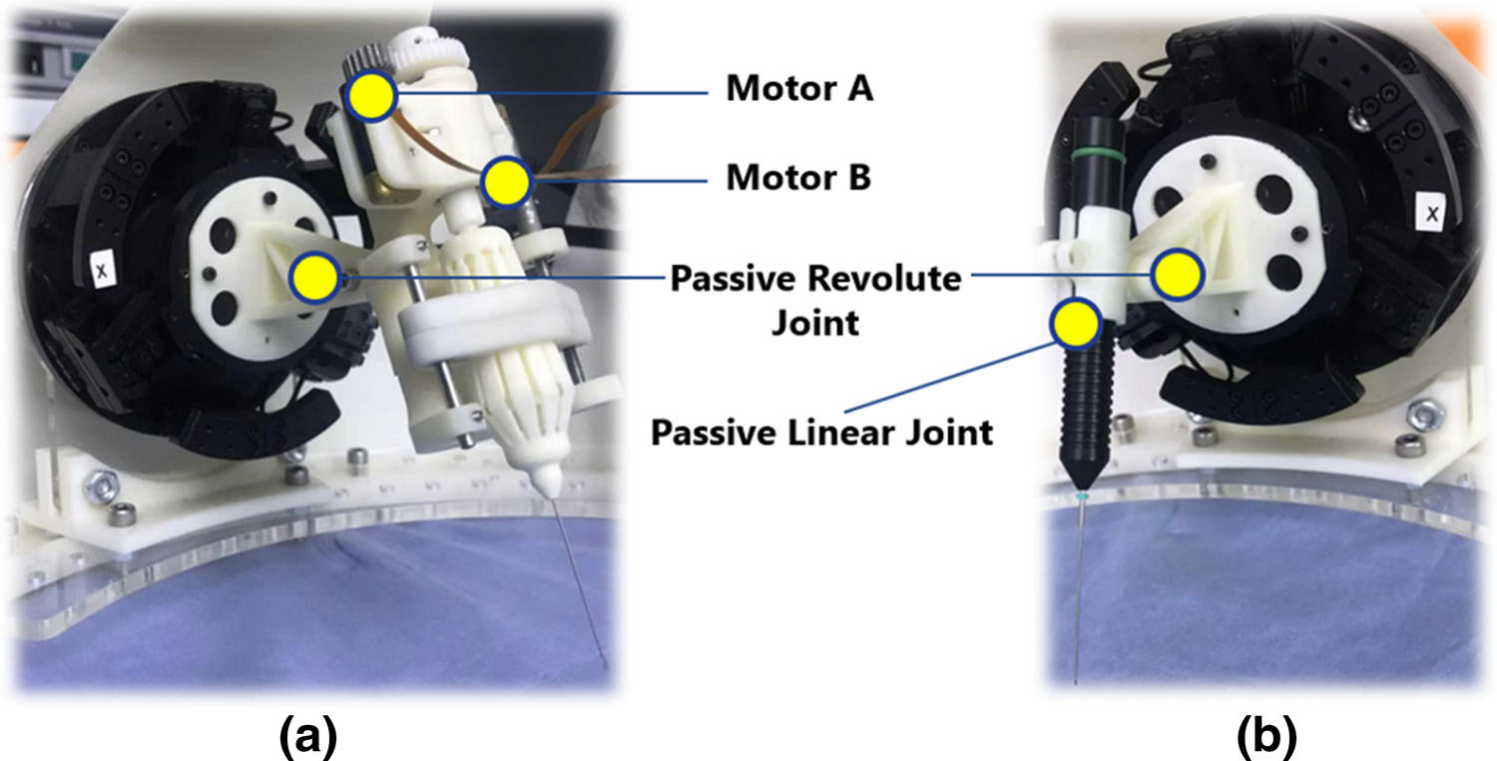
Physical prototypes of microsurgical tools which are mounted on the manipulators of the slave robot. **a** The motorized microforceps; **b** the micropolisher with adjustable assembled angle

Figure 3a shows the prototype of a motorized 25ga Maculorhexis Forceps (Katalyst Surgical, LLC, USA). Two brushless DC motors are used for active control of the microforceps. Figure 3b is a 25ga Tano Polisher (Katalyst Surgical, LLC, USA), which is used for neuroepithelial membrane polishing. The relative position of the tool can be adjusted via a passive revolute joint and a passive linear joint. Users can replace the current set-up with other types of microsurgical tools with ease (by mounting them on the slave robot via a customized 3D-printed connector for different applications).

#### Vision system

**Fig. 4:**
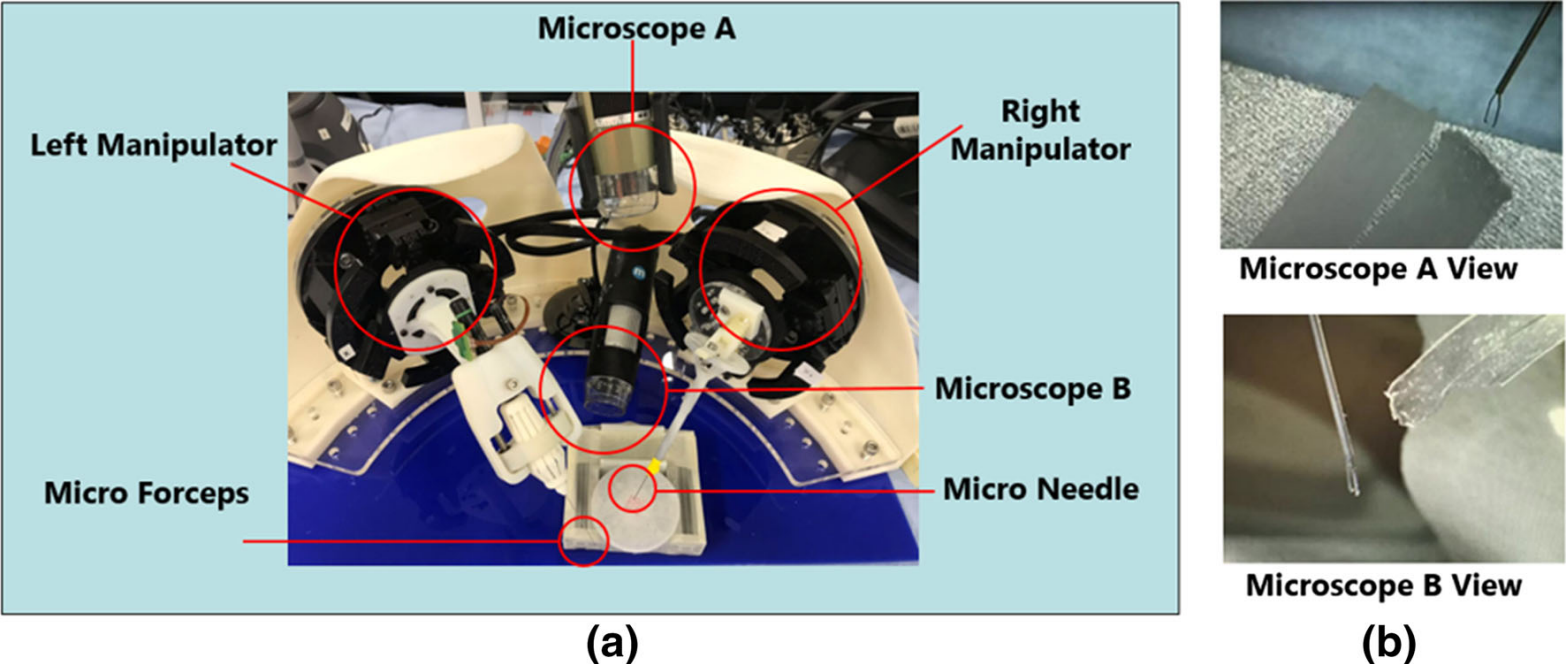
MRRP slave robot and the views from the digital microscopes. **a** Overview of the MRRP slave robot; **b** the digital microscopes’ views for microsurgical operation

An overview of the proposed MRRP slave robot with microsurgical tools is shown in Fig. 4a. For the vision system, two microscopes are utilized to monitor the operation scenes. A digital microscope (Dino Lite, UK) towards the top plate of the positioning stage is employed to provide 2D visual feedback during remote control. An example view of the microscope is shown in Fig. 4b (Microscope A View). Another digital microscope (Maplin n43HH, UK) is used for depth perception, the view of which is indicated in Fig. 4b (Microscope B View). The magnification of both microscopes is adjustable.

#### The master control console

In general, master controllers for microsurgery include two categories, i.e. grounded master manipulator and hand-held master controller. For the MRRP, Phantom Omni (Geomagic Touch, USA) is employed as a grounded master manipulator. The pen-like end-effector of the Phantom Omni can enable the operator to have intuitive control of the microsurgical tool through teleoperation [16].

As an alternative, an ungrounded controller has inherent advantages [17]. For example, it is easy-to-use, lightweight and more natural, since there are no constraints for motions. Therefore, a bespoke hand-held master controller is developed based on visual and inertial motion tracking. More detailed illustrations are described in [18].

**Fig. 5:**
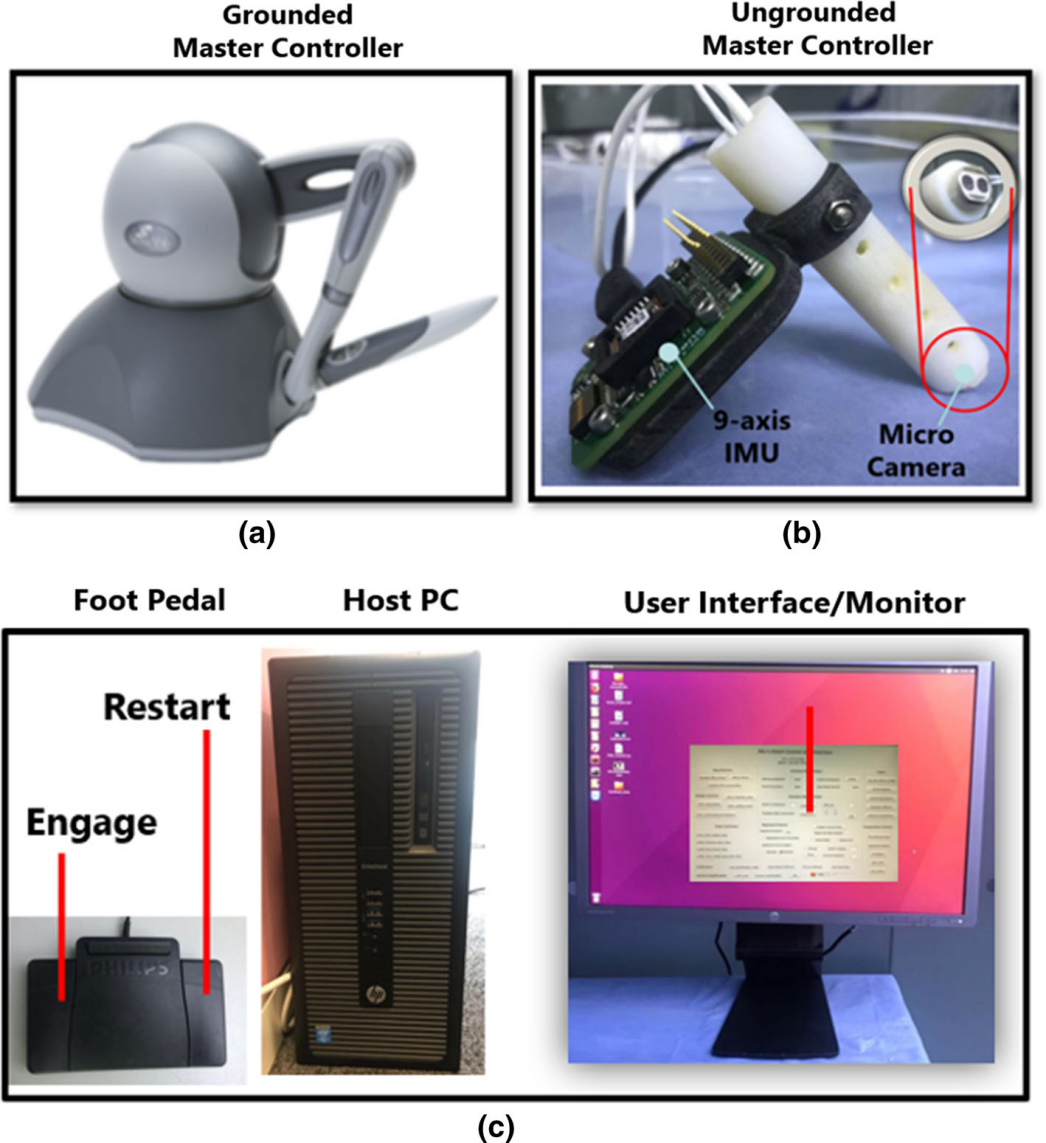
Overview of the master control console. **a** Grounded master manipulator (Phantom Omni); **b** ungrounded master manipulator (an in-house hand-held controller); **c** the overview of other components for the master control console

The master control console for the MRRP can be viewed in Fig. 5, which includes the grounded master manipulator (Fig. 5a) and an ungrounded master manipulator (Fig. 5b). Figure 5c indicates the display system with a graphical user interface (GUI), the host PC and the foot pedal (Philips LFH2310) for providing ‘engage’ and ‘clutch’ commands during teleoperation. The images captured by the two digital microscopes can be visualized on the monitor, so the operator can conduct remote control of the slave robot with ease based on the real-time visual feedback.

**Fig. 6:**
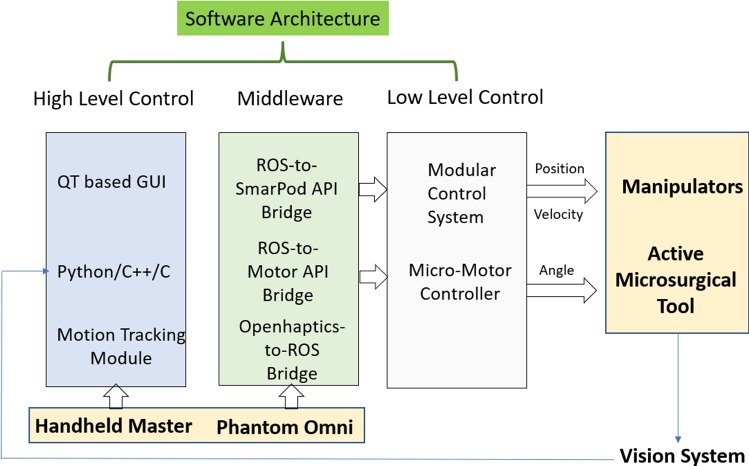
Overview of the software architecture for MRRP

**Fig. 7:**
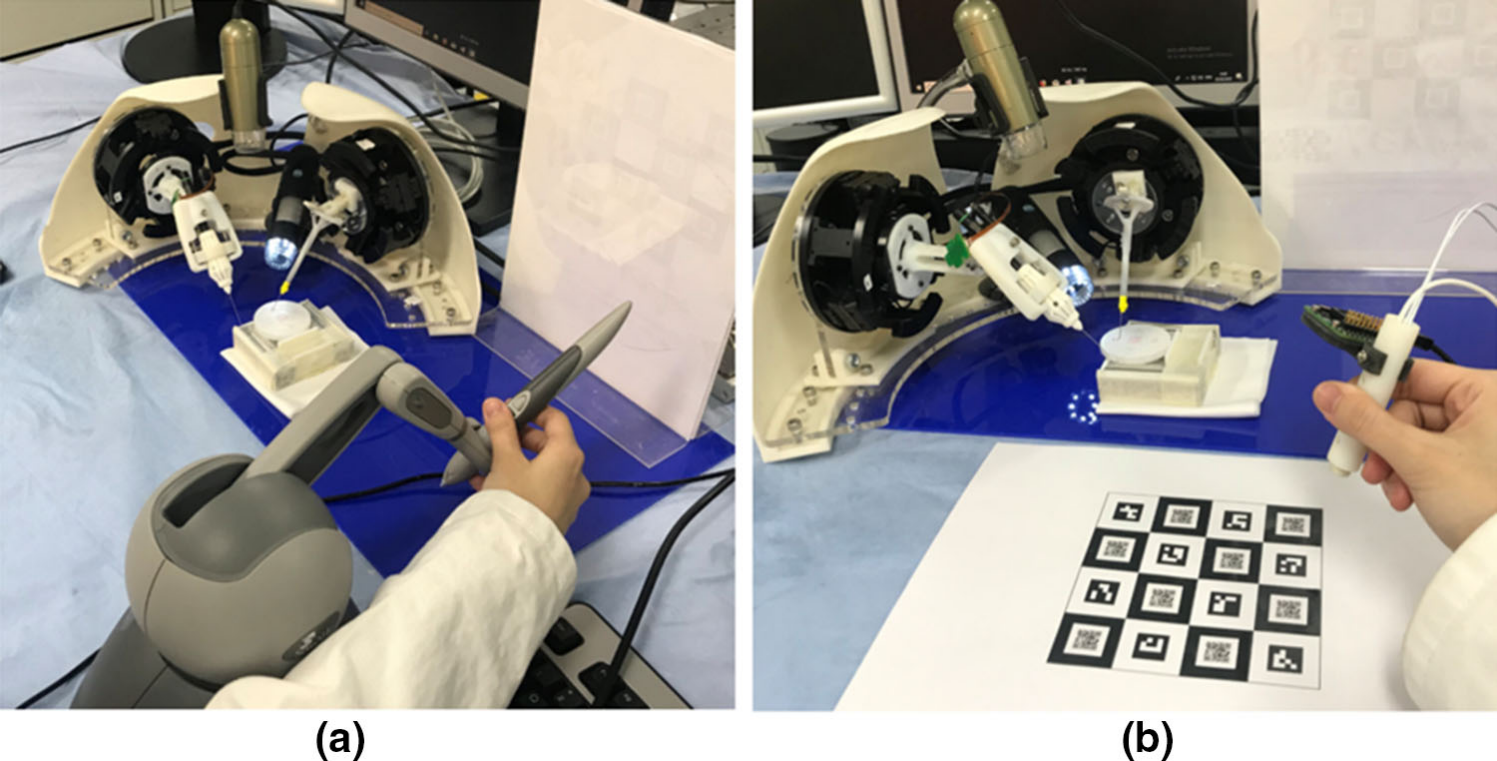
Scenes of the microsurgical robot control. **a** Operation using the Phantom Omni. **b** Operation using the hand-held controller

### Software architecture

An overview of the software architecture for MRRP is illustrated in Fig. 6. The kinematic control of the slave robot is based on the SmarPod API. A modular control system (SmarAct, Germany) is used to control the piezomotors of the slave robot manipulators, while two other motor controllers are utilized to control two brushless DC motors (Faulhaber) for the motorized microforceps, both of which form the low-level control system. The modular control system for the manipulator can have a sampling rate of 1 kHz for trajectory control.

In our proposed system, the ROS is used as a middleware to interface with the MRRP. We developed a ROS-to-SmarPod API Bridge with components that publish the robot’s states as ROS messages. The real-time kinematic and visual data of the robot can be subscribed as ROS messages (topics) for additional high-level processing.

In the meantime, the control commands generated by the master manipulators via the human operators or an intelligent system can be published as ROS topics to set the MRRP robot’s end-effector at the targeted pose in the Cartesian Space. With the proposed ROS-to-Smarpod API Bridge, the high-level commands and the low-level motor control interface can be linked via the ROS-to-Smarpod API Bridge.

The OpenHaptics toolkit is developed to ease the incorporation of haptics [19]. To interact with the microsurgical robot with the Phantom Omni device, an OpenHaptics-to-ROS Bridge is employed to link the OpenHaptics toolkit and the Phantom Omni. As for the hand-held master controller, the operational commands are generated by a motion tracking module based on OpenCV, and to control the microsurgical robot via the ROS-to-Smarpod API. All the high-level calculation and processing can be implemented through Python, C++ and C programming languages, while the QT-based GUI enables the development of intuitive user interfaces.

### System integration

Since the MRRP is constructed based on master–slave control, the mapping relationship between the master robot and the slave robot should also be considered.

When operators are using the master-side manipulator to finish a given task, two modes of control methods can be chosen: (1) position mapping mode and (2) velocity mapping mode. The definitions of the two mapping modes are shown as follows.

Here, we denote $\varvec{P_\mathrm{s}(t)}$ and $\varvec{P_\mathrm{m}(t)}$ as the slave robot and the master robot’s end-effector position at time step *t*, respectively, $\tau$ as the motion scaling factor, ${\Delta }t$ as the time interval for control, and $\varvec{V_\mathrm{m}(t-1)}$ as the master robot’s end-effector velocity. We can have the following mapping modes.

- **Position mapping mode**: mapping the position change of the master to the slave by a scaling factor, for more precise control of the end-effector with fine movements:
  1 $$\begin{aligned} \varvec{P_\mathrm{s}(t)} = {\tau }(\varvec{P_\mathrm{m}(t)} - \varvec{P_\mathrm{m}(t-1)}) + \varvec{P_\mathrm{s}(t-1)} \end{aligned}$$
- **Velocity mapping mode**: mapping the displacement and direction from the operation centre of the master device to the magnitude and direction of velocity of the slave side robot, which offers a non-clutching mapping for coarse movement.
  2 $$\begin{aligned} \varvec{P_\mathrm{s}(t)} = \varvec{V_\mathrm{m}(t-1)}{\Delta }t + \varvec{P_\mathrm{s}(t-1)} \end{aligned}$$

After the integration of the software architecture and the hardware system with appropriate master–salve mapping, the microsurgical training can be conducted using the Phantom Omni or the hand-held master controller as the control interface, as shown in Fig. 7. A comparative study for using these two types of master controller has been presented in [18].

## A novel hybrid interface

In this section, a novel hybrid interface is proposed, while the evaluation is conducted based on the MRRP for research purposes. Comparisons are made between the anisometric interface and the hybrid interface.

### A hybrid interface

Position mapping mode is the most common mapping strategy for teleoperation. For position mapping, a clutching mechanism is normally required to reposition the master manipulator when it reaches its workspace boundary during teleoperation. This may influence the consistency of surgical procedures. Velocity mapping mode can be implemented to enhance the operation efficiency by eliminating the clutching number [20]. However, the accuracy of velocity mapping is not high enough for the operators to fulfil the whole microsurgical procedures with ease. Since the two mapping modes have relative advantages and disadvantages, a more versatile interface is necessary [21]. To this end, a novel hybrid interface is proposed to combine their respective advantages.

An anisometric interface has been used in existing surgical robot control, which employs displacement as control input [22]. The isometric interface utilizes the force or torque as the control input, which does not allow large movements from the operator. The definitions of the two interfaces are shown as follows:

- **Anisometric Interface**: The control input is the master end-effector displacement, while the control output is the position of the end-effector of the slave robot;
- **Isometric Interface**: The control input is the applied force or torque for the master manipulator, while the control output is the velocity of the end-effector of slave robot.

The anisometric interface can ensure high precision control, and it is based on the position mapping mode. The isometric interface does not allow large movements from the operator, which can reduce the number of clutching. A hybrid interface developed by integrating the anisometric interface and the isometric interface may enhance the control efficiency while retaining the high precision control required for the RAMS. Therefore, the proposed hybrid control interface is realized by switching between the anisometric interface for position control and the isometric interface for velocity control.

By pressing the two buttons on the Phantom Omni, the anisometric interface and the isometric interface can be switched between each other. The control of the anisometric interface is the same as the position mapping mode. As for the control of the isometric interface, the initial velocity is set to zero at the mapping mode switching point, a virtual spring is used as feedback, so that the user can feel how velocity is commanded. The velocity control vector is proportional to the master position increment generated by the operator. During switching between the two interfaces, the ‘clutch’ mechanism works automatically to ensure stability.

When the operator presses the two buttons of the Phantom Omni, the end-effector of the slave robot will remain in the same place and enter the ‘lock’ status, where the position of the end-effector is $\varvec{P_\mathrm{l}}$. At time step *t*, the updated end-effector position controlled by the user is $\varvec{P_\mathrm{m}(t)}$. The end-effector of the slave robot can be calculated by (3), where *k* is a user-defined parameter. For all the user studies conducted in this paper, *k* is set to be 0.04.

3 $$\begin{aligned} \varvec{P_\mathrm{s}(t)} = {k}(\varvec{P_\mathrm{m}(t)} - \varvec{P_\mathrm{l}}){\Delta }t + \varvec{P_\mathrm{s}(t-1)} \end{aligned}$$

The hybrid control method is developed by exploiting the advantages of both mapping methods. When the operator is moving towards a distant target, the motion can be regarded as coarse motion. Therefore, the velocity mapping mode can be used to accelerate the speed of movement. For local operation such as positioning the targeted point, the control interface can be switched to the position mapping mode to ensure accuracy.

### User studies

In order to verify the effectiveness of the interface developed, two user studies were conducted. The user studies include a trajectory following task and a targeting task.

**Fig. 8:**
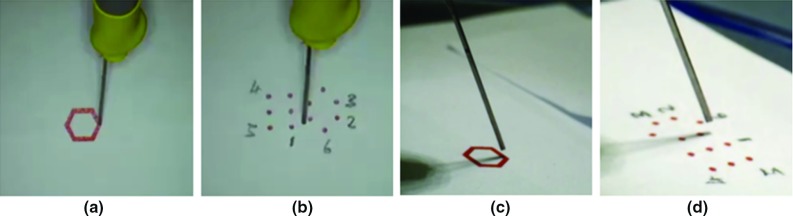
Scenario for user studies. **a** Visual feedback from Microscope A for trajectory following task. **b** Visual feedback from Microscope A for targeting task. **c** Visual feedback from Microscope B for trajectory following task. **d** Visual feedback from Microscope B for targeting task

For the trajectory following task, subjects were required to follow a red hexagon path and place their tooltip as close to the reference trajectory as possible. In this way, the positional manoeuvres can be evaluated. The trajectory for tracking is illustrated in Fig. 8a, while Fig. 8c shows the side view for depth monitoring.

As for the targeting task, subjects were required to point the microneedle tooltip to the targeted point precisely within a short tolerant distance based on a pre-defined protocol. Six points were selected as targets (see Fig. 8b), while Fig. 8d shows a side view for depth monitoring.

Eight subjects were recruited for the user studies. The test began after all the subjects finished the practice section. Both kinematic data and video data were collected. Each subject performed three trials for each interface, i.e. (i) anisometric interface and (ii) hybrid interface. Since the velocity mapping mode has been proved to be imprecise based on the user study results, the comparison of this mode is not included in this paper [20]. A total of 48 trials were conducted.

### Results and analysis

Performance evaluation for the user studies includes task completion time for a single trial (CT), the average velocity of the slave robot for finishing each trial (AV), the total path length of the slave robot end-effector trajectory (ST) and that of the master controller (MT), the number of clutching (CN), and the control efficiency (CE). CE represents the ratio of the total path length of the slave robot end-effectors and that of the master manipulators, which reveals how efficiently the operation’s motions are mapped to the slave robot.

The bigger the value of CN, the longer the time for repositioning the master manipulator, which contributes to a higher value of MT. As for the slave robot, it keeps still during clutching, so the value of ST will not be influenced by CN significantly. More detailed illustration of the evaluation metrics for master–slave mapping can be found in [23].

**Table 1:** User study results for the trajectory following and the targeting task

Metrics	Anisometric interface	Hybrid interface	SW test	*p* value
Trajectory following				
ST (m)	$0.015\pm 0.002$	$0.016\pm 0.004$	9.62E$-$09	0.3271
CT (s)	$42.218\pm 18.633$	$39.559\pm 15.682$	0.0277	0.5953
AV (mm/s)	$0.427\pm 0.194$	$0.455\pm 0.188$	0.0012	0.6179
MT (m)	$2.005\pm 0.314$	$1.485\pm 0.535$	0.4695	0.0004
CN	$16.8\pm 6.6$	$9.0\pm 6.3$	0.1271	0.0001
CE	$0.008\pm 0.001$	$0.012\pm 0.005$	7.57E$-$07	4.23E$-$05
Targeting				
ST (m)	$0.025\pm 0.002$	$0.026\pm 0.002$	0.1355	0.0002
CT (s)	$39.607\pm 15.199$	$39.605\pm 12.275$	0.0593	0.5485
AV (mm/s)	$0.725\pm 0.290$	$0.732\pm 0.184$	0.0017	0.9243
MT (m)	$3.334\pm 0.371$	$1.984\pm 0.816$	0.0030	2.49E$-$09
CN	$23.5\pm 9.5$	$11.7\pm 8.1$	0.0274	2.97E$-$05
CE	$0.008\pm 0.001$	$0.016\pm 0.006$	5.12E$-$07	6.85E$-$08

Normality tests (Shapiro–Wilk test) at 0.05 significance level were performed before detailed statistical analysis. Since all the subjects completed repetitions of the two control interfaces, the user studies are within-subject design, where Wilcoxon signed-rank tests are applied for nonparametric statistical comparison between variables, while *T* tests are used for the other evaluation metrics.

#### Trajectory following task

**Fig. 9:**
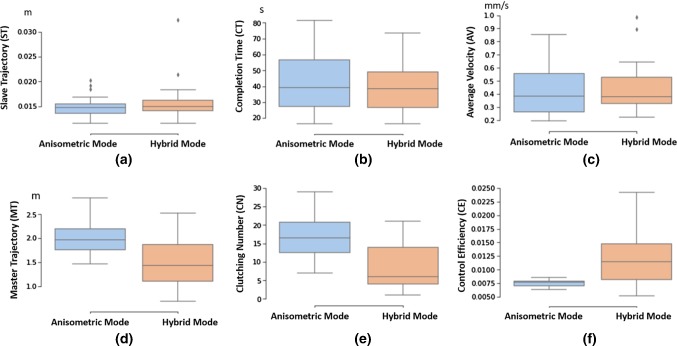
Box plot for user studies’ results of the trajectory following task. Comparisons in terms of **a** slave robot trajectory (ST); **b** task completion time (CT); **c** average speed (AV); **d** master robot trajectory (MT); **e** clutching number (CN); **f** mapping efficiency (ME)

Based on the user study results (see Table 1), we can see that the hybrid interface has better performance in terms of all the evaluation criteria for the trajectory following task, except for ST. The box plot results are shown in Fig. 9. A *p* value $<0.05$ is considered significant.

For the trajectory following task, the obtained experimental data of the performance metrics (CN and MT) reveal a nonparametric distribution, while the data of other metrics satisfy the normal distribution assumption. Based on the *p* value, the differences between the slave robot trajectory and the task completion time, as well as the average control speed, are not significant (*p* value $>0.05$).

The total path length of the master for the trials was significantly shorter for the hybrid interface compared to that of the anisometric interface (1.485 m vs. 2.005 m, ${p} = 0.0004$). Using the hybrid interface, average reduced clutching was observed (9.0 vs. 16.8, ${p}=0.0001$), while the subjects have significantly higher control efficiency (0.012 vs. 0.008, ${p} = 4.23\hbox {E}-$05).

#### Targeting task

**Fig. 10:**
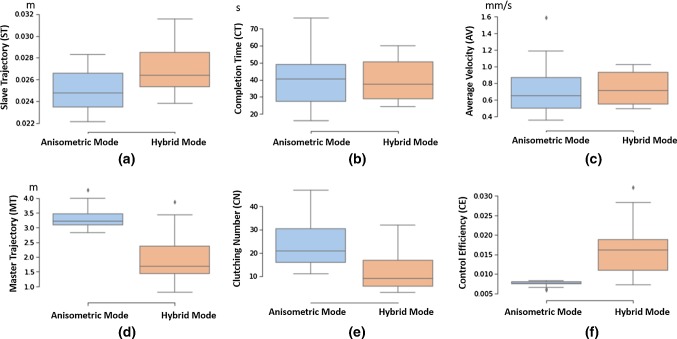
Box plot for user studies’ results of the targeting task. Comparisons in terms of **a** slave robot trajectory (ST); **b** task completion time (CT); **c** average speed (AV); **d** master robot trajectory (MT); **e** clutching number (CN); **f** mapping efficiency (ME)

As for the targeting task, except for the anisometric interface, showing shorter total path length of the slave robot, the task completion time is similar for both modes. The hybrid interface has a higher value of average speed for task completion with a significantly reduced number of clutching, a shorter master robot path and enhanced control efficiency. The corresponding box plot results are provided in Fig. 10.

The obtained experimental data of evaluation metrics (ST and CT) reveal a nonparametric distribution. The differences between the task completion time and the average control speed are not significant (*p* value $>0.05$).

In terms of the clutching number involved, the value of the hybrid interface was significantly smaller than that of the anisometric interface (11.7 vs. 23.5, ${p}=2.97\hbox {E}-$5), which contributed to a smooth operational workflow. This is promising, as the subjects can improve the control efficiency by nearly 50% (0.016 m vs. 0.008 m, ${p} = 6.85\hbox {E}-$08), reducing more than 30% of the total path length of the master manipulators (1.984 m vs. 3.334 m, ${p} = 2.49\hbox {E}-$09), due to the avoidance of constant repositioning.

## Discussion

This paper proposed a microsurgical telerobotic platform MRRP for exploratory research. Research on the hybrid interface further confirms its potential for assisting research on microsurgical robotics. The user studies carried out in this work show that the targeting and the trajectory following tasks can be accomplished through master–slave control.

However, more complex microsurgical tasks can be proposed to evaluate the effectiveness of the platform. For example, it may be useful to investigate other tasks such as orientating and membrane peeling. An orientating task can be realized by changing the slave manipulator posture while maintaining the tip position. For example, the subject can align the needle with the horizontal line starting from the line with a specific angle [24]. Membrane peeling can be achieved through microforceps to manipulate artificial membrane.

As for the hybrid interface, the effectiveness of the hybrid interface has been verified. However, the switching between two mapping modes is decided by the operator manually. The number of transitions between modes and the percentage of test time belonging to position mapping mode and the velocity mapping mode is determined by users’ preferences and operational habits. Further introduction of context-awareness of the microsurgical scenes can assist the implementation of the intelligent hybrid interface with automatic switching [23]. Therefore, future work will include developing an adaptive hybrid interface to further enhance the teleoperation efficiency for microsurgical robot remote control.

## Conclusions and future works

In summary, we have presented a ROS-based MRRP in this paper for RAMS research and training. The slave robot of MRRP is developed based on a 6-DoF hexapod positioning stage, while Phantom Omni (a commercial haptic device) and an in-house hand-held master controller are used as the grounded and ungrounded master manipulators of the control console, respectively. A software architecture for the MRRP is developed based on ROS.

A hybrid interface for MRRP is proposed by switching between the isometric interface and the anisometric interface to enhance the mapping efficiency for RAMS. Experimental validation has been conducted to prove the usability of the platform and the proposed interfaces. The results of the user studies indicate that the proposed hybrid interface is advantageous in terms of reduced number of clutching and shorter path length of the master controller end-effector, thus improving the overall control efficiency. In summary, the MRRP can serve as a versatile platform for research development and microsurgical skill training.
